# 

**Published:** 1902-12

**Authors:** 


*THE BRISTOL MEDICO-CHIRURGICAL SOCIETY.*

CONTENTS.

| | PAGE |
|---|---|
| **The Medical Life.** By GEORGE MUNRO SMITH, M.R.C.S., L.R.C.P. ... ... ... | 289 |
| **The Results of Operation in Fifty-Four Cases of Cancer of the Breast.** By CHARLES A. MORTON, F.R.C.S. ... ... | 305 |
| **The Indications for Treatment in Nephroptosis.** By JAMES SWAIN, M.S., M.D. Lond., F.R.C.S. ... ... ... | 315 |
| **Observations on Cases of Appendicitis.** By T. CARWARDINE, M.S.Lond., F.R.C.S. | 319 |
| **Tubal Abortion.** By D. C. RAYNER, F.R.C.S. | 323 |
| **Vasomotor and Ocular Phenomena in Relation to Rheumatoid Arthritis.** By R. LLEWELYN JONES, M.B. Lond., M.R.C.S., L.R.C.P. ... ... ... | 328 |
| **Progress of the Medical Sciences:** | |
| **Medicine.** J. MICHELL CLARKE, M.A., M.D. Cantab., F.R.C.P. ... ... ... ... | 333 |
| **Surgery.** JAMES SWAIN, M.S., M.D.Lond., F.R.C.S. ... ... ... ... ... ... ... | 336 |
| **Otology.** W. H. HARSANT, F.R.C.S. ... | 340 |
| **Laryngology and Rhinology.** P. WATSON WILLIAMS, M.D. Lond. ... ... ... ... | 342 |
| **Reviews of Books** ... ... ... ... ... ... ... | 345 |
| **Editorial Notes** ... ... ... ... ... ... ... | 364 |
| **Notes on New Drugs and Preparations for the Sick** ... ... ... ... ... ... | 368 |
| **The Library of the Bristol Medico-Chirurgical Society** ... .. ... ... | 372 |
| **Meetings of Societies** ... ... ... ... ... | 377 |
| **Local Medical Notes** ... ... ... ... ... | 382 |

